# Leptin and the homeostatic system regulating body weight

**DOI:** 10.1186/1753-6561-6-S3-O6

**Published:** 2012-06-01

**Authors:** Jeffrey M Friedman

**Affiliations:** 1 Howard Hughes Medical Institute, the Rockefeller University, New York, NY 10021, USA

## 

The discovery of leptin has led to the elucidation of a robust physiologic system that maintains fat stores at a relatively constant level. Leptin is a peptide hormone secreted by adipose tissue in proportion to its mass. This hormone circulates in blood and acts on the hypothalamus to regulate food intake and energy expenditure. When fat mass falls, plasma leptin levels fall stimulating appetite and suppressing energy expenditure until fat mass is restored. When fat mass increases, leptin levels increase, suppressing appetite until weight is lost. By such a mechanism total energy stores are stably maintained within a relatively narrow range.

Recessive mutations in the leptin gene are associated with massive obesity in mice and some humans. Treatment with recombinant leptin markedly reduces food intake and body weight. The low leptin levels in patients with leptin mutations are also associated with multiple abnormalities including infertility, diabetes and immune abnormalities all of which are corrected by leptin treatment. These findings have established important links between energy stores and many other physiologic systems and led to the use of leptin as a treatment for an increasing number of other human conditions including a subset of obesity, some forms of diabetes including lipodystrophy and hypothalamic amennorhea, the cessation of menstruation seen in extremely thin women. Identification of a physiologic system that controls energy balance establishes a biologic basis for obesity and further establishes links between leptin and numerous other physiologic responses. Recent studies have explored the relationship between leptin and the reward value of food. In addition, new methods for identifying neurons activated by leptin and other stimuli have been developed.

